# A randomised controlled trial of the effectiveness of self-weighing as a weight loss intervention

**DOI:** 10.1186/s12966-014-0125-9

**Published:** 2014-10-10

**Authors:** Claire D Madigan, Kate Jolly, Amanda L Lewis, Paul Aveyard, Amanda J Daley

**Affiliations:** Health and Population Sciences, College of Medical and Dental Sciences, University of Birmingham, Birmingham, B15 2TT UK; School of Social and Community Medicine, University of Bristol, Canynge Hall, 39 Whatley Road, Bristol, BS8 2PS UK; Department of Primary Care Health Sciences, University of Oxford, Radcliffe Observatory Quarter, Woodstock Road, Oxford, OX2 6GG UK

**Keywords:** Weight loss, Obesity, Self-weighing, Self-monitoring

## Abstract

**Background:**

There is a need to find simple cost effective weight loss interventions that can be used in primary care. There is evidence that self-monitoring is an effective intervention for problem drinking and self-weighing might be an effective intervention for weight loss.

**Purpose:**

To examine the efficacy of daily self-weighing as an intervention for weight loss.

**Methods:**

A randomised controlled trial of 183 obese adults, follow-up three months. The intervention group were given a set of weighing scales and instructed to weigh themselves daily and record their weight. Both groups received two weight loss consultations which were known to be ineffective.

**Results:**

92 participants were randomised to the intervention group and 91 to the control group. The intervention group lost 0.5 kg (95% CI 0.3 to 1.3 kg) more than the control group, but this was not significant. There was no evidence that self-weighing frequency was associated with more weight loss.

**Conclusions:**

As an intervention for weight loss, instruction to weigh daily is ineffective. Unlike other studies, there was no evidence that greater frequency of self-weighing is associated with greater weight loss.

**Trial registration:**

ISRCTN05815264

## Background

Primary care physicians potentially have a big role in addressing obesity in their patients, but they exercise this role uncommonly [1,2]. One key reason is that physicians do not believe that such interventions are effective [3]. Evidence from observational studies suggests that it may be helpful if they were to raise the topic of excess body weight [4,5]. It is possible that simply prompting weight loss may be sufficient to motivate some people to attempt weight loss and succeed, however other techniques may be more effective.

We know that referral and participation in multi component weight loss interventions are effective for weight loss, however not all people attend these programmes [6-9]. Offering brief interventions in a primary care setting may reach more people and be cheaper for health services. One promising technique is to suggest a patient weighs themselves daily and record their weight. A meta-regression of interventions for reducing problem drinking found that encouraging participants to record their daily consumption of alcohol appeared to explain most of the variation in effectiveness of brief interventions [10]. There is also evidence that self-monitoring is a brief effective technique for healthy eating and increasing physical activity [11]. Multi component randomised controlled trials (RCTs) focused on self-weighing resulted in significant weight loss compared to a control group that did not self-weigh [12,13]. However there have been only two RCTs that have isolated the effect of self-weighing and these found no significant differences at programme end, although they were very small and had high attrition which makes interpretation difficult [14,15]. Self-weighing has also been shown to be effective for weight loss maintenance [16].

Based on the self-regulation theory, self-weighing can show the individual how their energy intake and expenditure affects their weight [17]. For self-weighing to be effective, people have to be able to reflect on how their behaviour has affected their weight, make plans to change that behaviour, and enact those plans. It is possible, then, that we may need to add other techniques or components to self-weighing incrementally to build an effective yet simple to deliver brief intervention for primary care use.

The aim of this trial is to start intervention building. We examined this in an explanatory trial to test whether asking patients to weigh themselves daily would help them lose weight, before testing it as an opportunistic brief intervention. This involved giving extra support to participants to ensure they did weigh themselves regularly. It also involved creating a “sham” weight loss treatment for the control group that aimed to motivate their continued weight loss attempt and adherence to follow-up.

## Methods

### Design

Two arm individually randomised trial with blinding of the participants and those conducting follow-up. Participants were allocated to the intervention group of self-weighing or control group. Ethical approval was given by NRES Committee West Midlands, England 31/05/2012 Reference: 12/WM/0137.

### Participants

Two family practices within England agreed to participate. A total of 1914 patients with a raised BMI (≥30 kg/m^2^) recorded within their primary care medical notes in the past 15 months were invited to take part, by letter from their family practitioner. Patients completed a screening questionnaire by telephone or sent this back by post and eligible participants were given an appointment at their family practice.

### Inclusion criteria

Participants were aged ≥18 years with a raised BMI of ≥30 kg/m^2^.

### Exclusion criteria

Participants were excluded if they: were pregnant or intending to become pregnant; could not understand or speak English sufficiently to undertake the tasks of the study; were currently attending a weight loss programme (including pharmacotherapy or bariatric surgery) or had taken part in a formal weight management programme in the previous three months. They were also excluded if they reported weighing themselves at least once per week as the intervention aimed to get people who do not regularly weigh themselves to do so and use this feedback to take action to control their weight.

### Sample size

Based on the assumption that the intervention group would lose 1.0 kg (SD 2.0 kg) more than the control group at follow-up with 80% power and 5% type I error, 180 participants were required (including allowing for losing 30% at the three month follow-up). We chose a small difference of 1.0 kg as worth detecting, considering the minimal nature of the intervention. The SD was taken from a similar study of a low intensity primary care weight loss intervention [18].

### Allocation and randomisation

Participants were randomised after eligibility assessment and consent was taken. An independent statistician prepared random block sizes of between two and eight to ensure balance of trial arms. Researchers obtained informed consent from patients and then, using opaque sealed envelopes, randomly allocated participants to their treatment group.

### Blinding

Participants were blinded to group allocation, i.e. neither group was told that this was a trial about self-weighing; the information sheet informed patients that it was a trial about losing weight. Independent researchers measured participants’ weight at three months.

### Settings

Both groups received weight loss consultations at their family practice by a researcher. Three month follow-up took place either at the family practice or at the participant’s home.

### Components across both groups

We wanted to isolate the effect of self-weighing by giving no effective intervention except self-weighing but sought to maintain blindness to the real purpose of the trial and to minimise follow-up bias by giving the control group a plausible yet ineffective intervention. This type of intervention is similar to that a family practice nurse might deliver and, in a more intensive form, has been shown to be ineffective [6]. Both groups received this same intervention which consisted of two visits to the family practice. At visit one (after randomisation at the same visit) participants received a 45 minute consultation to discuss weight loss tips (components can be found in Table 1), a booklet titled ‘Your Weight, Your Health’ and a basic four-day food diary that was to be completed before the next visit, seven days later (visit 2) [19]. At visit two participants discussed the completed food diary with the researchers. Participants were advised they should aim to lose 0.5 kg of body weight per week in line with NICE guidance in England [20].

**Table 1:** **Behavioural change techniques used in the intervention based on CAL-ORE taxonomy** [23]

**Behavioural technique**	**Definition**
**Intervention only**	
Prompt self-monitoring of behavioural outcome	Participants were instructed to weigh themselves daily and record it on the weight record card provided.
Prompt review of outcome goals	Participants were instructed to work out their average weight for the week and review their progress against losing 0.5 kg per week.
Provide information on the consequences of behaviour in general	The benefits of self-weighing for weight loss were discussed with participants.
Environmental restructuring	Participants were asked to put the scales in a place that would help them remember to weigh themselves.
Provide information on where and when to perform the behaviour	Participants were asked to weigh themselves at the same time each day.
Use follow-up prompts	Participants were sent text messages once per week at a time which would help participants to remember to weigh themselves.
Barrier identification/Problem solving	At visit two participants were asked if there were any barriers to self-weighing and discussed how to overcome these barriers.
**Behavioural techniques given to both groups**	
Provide information about behaviour health link	The consequences of an unhealthy weight were discussed.
Provided general encouragement	Praised participants in week two for making changes to their diet and activity.
Goal setting (outcome)	Participants were instructed to lose 0.5 kg per week.
Prompt self-monitoring of behaviour	Participants were asked to complete a 4 day food diary.

### Intervention group

At visit one, participants were given weighing scales and instructed to weigh themselves daily and record their weight on the record card provided. On the record card at the end of each week there was a box participants could use to calculate their average weight for the week to compare to their target weight loss. Daily weighing was chosen over weekly weighing as immediate feedback on behaviour might institute the most effective learning and self-weighing is more likely to become habitual if it becomes part of a person’s daily routine [21,22].

As this was an explanatory trial we used behavioural techniques to help participants weigh themselves daily and are described in detail in Table 1. These techniques have been categorised based on the CALO-RE behavioural change taxonomy which is specific to changing diet and physical activity behaviours [23]. Briefly, the main technique used was self-monitoring of behavioural outcome by self-weighing. The benefits of self-weighing for weight loss were discussed and participants were instructed to aim for a weight loss of 0.5 kg per week and to review their average weight loss across the week against this target. Participants were told to weigh themselves at the same time every day to help self-weighing become a habit. They were also instructed to put the scales in a place which would help them remember to weigh themselves. Brief weekly text messages were sent to participants at times participants suggested were appropriate to prompt them to weigh themselves.

### Outcomes

The primary outcome was change in weight from baseline to three months. Secondary outcomes were physical activity and weight management strategies; we measured these as we hypothesised the behaviour of self-weighing should prompt a change in energy intake or expenditure based on the review of daily weight [24]. Diet was not measured as we wanted to reduce participant burden. Self-weighing frequency was measured objectively in the intervention group and was self-reported by both groups at baseline and three months by asking a single question: ‘how often do you usually weigh yourself?’ Due to technical failures the objective scale data was not available and we used participants’ daily record cards in the intervention group to record the frequency of weighing.

At baseline, participants reported socio-demographic data including: age, gender, ethnicity, postcode (converted to an index of multiple deprivation score [IMD]), occupation, medication and long-term health conditions [25]. IMD is an area-based measure of the socio-economic status and scores were categorised into quartiles [25]. Height was measured at baseline to the nearest centimetre and weight (kg) measured at baseline and follow-up on validated scales (SECA 875). If an objective measure of weight at follow-up could not be obtained self-reported weight was used. At baseline and follow-up, participants completed a questionnaire about weight management strategies they had used in the past month (adapted from a questionnaire previously used) and the international physical activity questionnaire (IPAQ-short) [26,27]. Physical activity was converted into MET minutes. Participants in the intervention group were asked on a Likert scale (1-9) if self-weighing affected their mood or made them change the way they felt about their body (a score of five being no difference) to identify any adverse effects. There was an open question where participants could provide comments about self-weighing.

### Data analysis

Continuous variables are shown as means and standard deviations or medians, and categorical variables as numbers and percentages. Descriptive data of age, gender and IMD were compared between those invited to take part and those who were randomised. All analyses were conducted using the intention to treat principle (ITT) and participants with missing weight data were assumed to have their baseline weight. Within group t-tests were used to examine if each group had lost a significant amount of weight between baseline and three months. The difference in weight change between the groups was analysed using linear regression. In a sensitivity analysis we adjusted for baseline variables to correct for any minor imbalances.

A post hoc analysis regression was used to examine the association between mean change in weight and the frequency of self-weighing adjusting for ethnicity, age and gender. We fitted both linear and quadratic terms. A quadratic term was fitted as we thought there may be a curved relationship between frequency of self-weighing and weight loss i.e. frequency of self-weighing may be useful up to a said frequency and would then result in the same amount of weight loss. We also counted the number of participants in the intervention group that calculated their average weight on their record chart across the week as another means of exploring engagement with the intervention.

Independent sample t-tests were used to examine differences between the group’s physical activity levels and change in hours spent sitting. Physical activity data were converted to MET minutes per week; one MET minute is defined as the resting metabolic rate when sitting at rest [28]. The mean change in weight management strategies used and confidence intervals were calculated.

## Results

**Figure 1:**
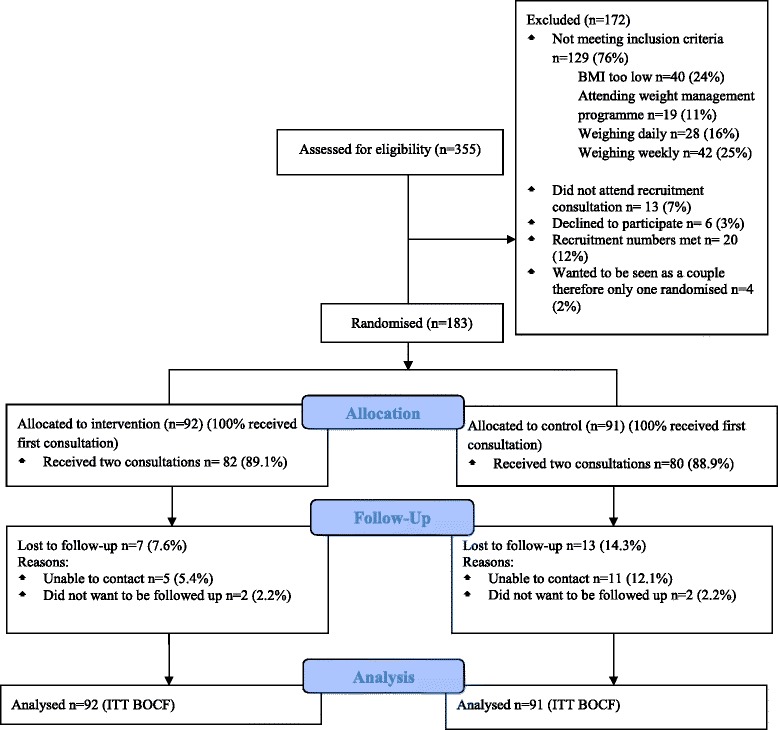
**CONSORT flow diagram.**

Participants were recruited between August and November 2012. In total 355 (18.5%) patients were assessed for eligibility (Figure 1). These were comparable in age, gender and IMD to those invited to take part by the family practices. Participants in both groups were similar on all baseline characteristics, although marginally more of the intervention group reported they had a long-term health condition (54.3 vs 42.9%) (Table 2). Follow-up rates were high at three months in both groups; 92.4% intervention group and 85.7% in the control group.

**Table 2:** **Baseline characteristics of participants**

	**Control**	**Intervention**
	**n = 91**	**n = 92**
Age mean (SD)	53.3 (14.6)	53.9 (14.9)
Male n (%)	33 (36.3)	34 (37.0)
Baseline BMI mean (SD)	36.2 (4.8)	35.8 (4.3)
White participants n (%)	59 (64.8)	60 (65.2)
Long-term health condition n = (%)	39 (42.9)	50 (54.3)
Taking medication n (%)	61 (67.0)	69 (75.0)
Deprivation*n (%)		
1 Highest deprivation quartile	68 (76.4)	70 (77.8)
2	15 (16.9)	17 (18.9)
3	6 (6.7)	2 (2.2)
4	0	1 (1.1)
Physical activity per week, MET minutes median (IQR)	744 (99 to 1740)	605 (177 to2079)

*Missing data for four participants, two in each group, all other variables n = 183.

### Primary outcome

The intervention group lost on average 0.5 kg (95% CI 0.3 to 1.3) more than the control group (non-significant, Table 3) and adjustment for covariates did not alter the results (data not shown). Both groups lost significant amounts of weight from baseline to three months. The control group lost 1.2 kg (95% CI 0.7 to 1.7) and the intervention group lost 1.7 kg (95% CI 1.1 to 2.3) (Table 3).

**Table 3:** **Analyses of weight change between baseline and three months**

	**Mean weight change baseline to follow-up**		**Mean difference between groups**
	**Control**	**Intervention**	**Unadjusted**
	**n = 91**	**n = 92**	**n = 183**
All participants followed up kg (95% CI)	-1.4 (-2.0 to -0.8)*	-1.8 (-2.5 to -1.1)*	-0.4 (-1.3 to 0.5) p = 0.4
Baseline weight observed carried forwards kg (95% CI)	-1.2 (-1.7 to -0.7)*	-1.7 (-2.3 to -1.1)*	-0.5 (-1.3 to 0.3) p = 0.24

Significance level *p <0.01.

### Adherence to self-weighing

Due to the exclusion criteria at baseline no participants reported weighing themselves daily or weekly. However using the single question about self-weighing at three months, 73.1% (n = 57 of 78 responses) of the intervention group reported weighing themselves at least once per week and of that, 60% (n = 47) weighed daily. Some of the control group also started to weigh themselves regularly with 19.4% (n = 14 of 72 responses) reporting weighing themselves at least once per week and of that, 11.1% (n = 8) weighed daily at follow-up. The weight record cards showed that, 21 (41%) of the participants in the intervention group calculated their average weight loss for the week at any time point. Fifty one (55%) participants who returned the weight record cards reported weighing themselves a median of 73 days (range 10 to 84).

**Figure 2:**
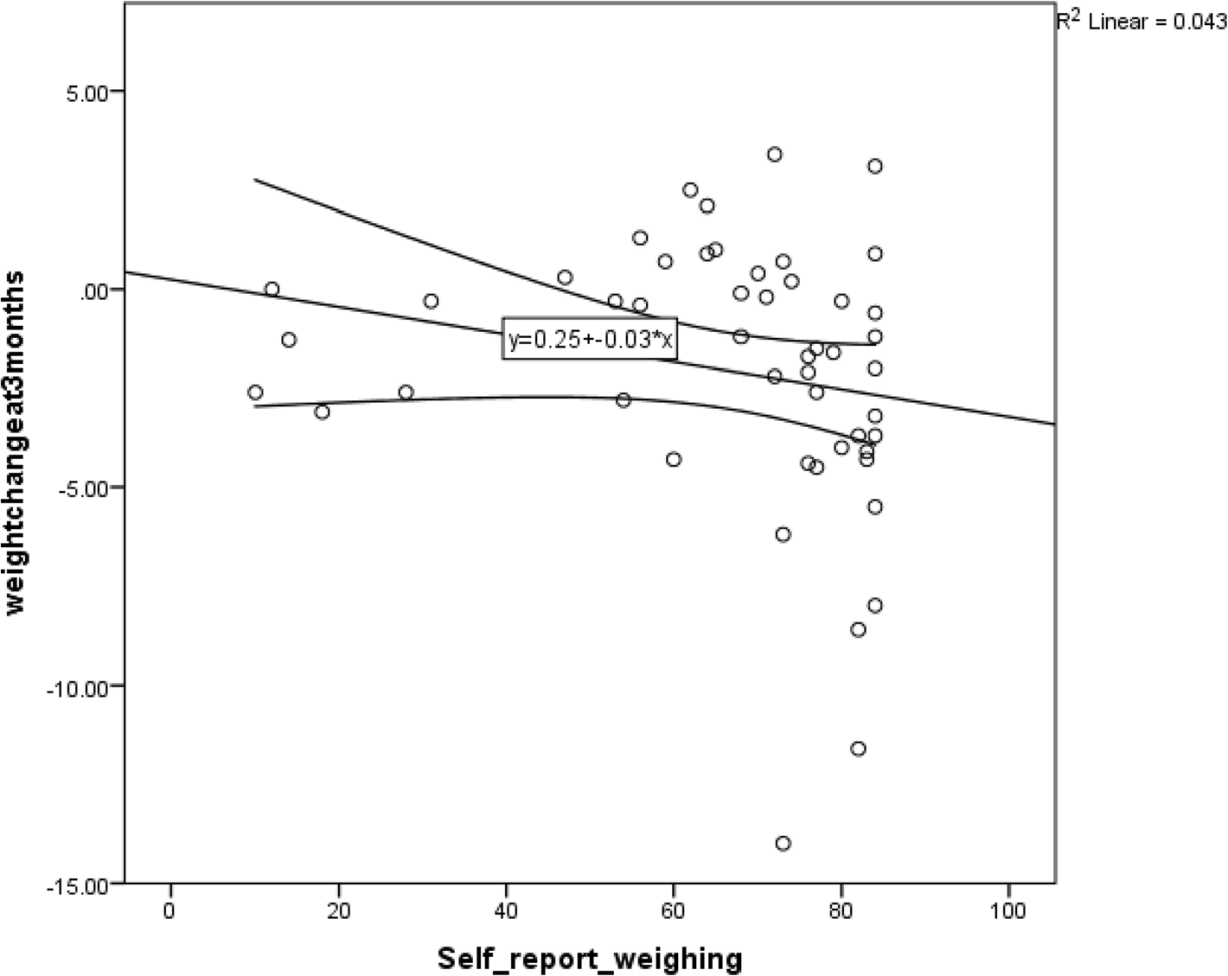
**Frequency of self-weighing and weight change with a line of best fit.**

We explored if frequency of self-weighing was associated with greater weight loss in the intervention group only. We fitted regression models of weight loss on frequency of self-weighing using linear and quadratic terms but as the quadratic term did not improve the fit it was omitted. There was no evidence that frequency of self-weighing was associated with greater weight loss, with each extra day of self-weighing associated with a 20 g (95% CI -30 to +20 g greater weight loss (Figure 2).

### Weight management strategies

We examined whether self-weighing prompted participants to use weight control strategies more frequently. There was limited evidence that it did; only the reported number of days keeping a record of what participants ate and drank increased significantly in the intervention group compared to the control group (mean difference 4.8 days/month 95% CI 1.3 to 8.2) (Table 4).

**Table 4:** **Mean number of days of weight loss strategies at baseline and three months**

**How often in the past month have you….**	**Mean change in control group (SD)**	**Mean change in intervention group (SD)**	**Mean changes between control and intervention (95% CI) days per month**
Planned your meals ahead of time?	2.3 (16.1)	0.8 (15.3)	-1.6 (-6.6, 3.5)
Tried to slow down your pace of eating?	4.7 (14.6)	7.4 (14.2)	2.6 (-2.0, 7.3)
Kept a record of what you eat and drink?	0.6 (10.6)	5.3 (10.7)	4.8 (1.3, 8.2)
Controlled your portions?	2.9 (13.7)	0.1 (15.3)	-2.8 (-7.5, 1.9)
Kept a goal for the amount of calories you eat per day?	1.5 (10.7)	2.4 (11.2)	0.8 (-2.7, -1.8)
Read nutrition labels?	-1.1 (16.1)	-0.2 (13.8)	0.9 (-3.9, 2.4)
Follow a consistent exercise routine?	1.0 (12.6)	-0.7 (12.8)	-1.8 (-5.9, 2.3)
Tried to limit eating out at restaurants?	2.2 (18.6)	-0.4 (17.5)	-2.6 (-8.4, 3.2)
Eaten breakfast?	2.1 (11.0)	0.6 (11.8)	-1.5 (-5.1, 2.2)
Chosen lower-calorie options of particular foods?	-0.8 (16.1)	1.8 (18.4)	2.6 (-3.0, 8.1)
Tried to avoid eating late at night?	1.4 (15.5)	0.8 (14.5)	-0.7 (-5.5, 4.2)
Tried to avoid doing other activities (e.g. watching TV) whilst eating?	3.5 (15.2)	2.7 (14.8)	-0.9 (-5.7, 1.7)
Do exercises that you enjoy?	0.3 (11.9)	-1.7 (11.1)	-2.0 (-5.7, 1.7)
Limited the amount of sugar you eat or drink?	3.3 (16.1)	-2.8 (17.7)	-6.1 (-11.5, -0.6)
Kept a goal for the amount of fruit and vegetables you eat per day?	-2.7 (16.7)	-0.3 (16.7)	2.3 (-3.1, 7.7)
Kept a goal for the grams of fat you eat per day?	1.9 (2.7)	3.8 (12.3)	2.0 (-2.1, 6.0)

### Physical activity

Self-reported physical activity levels were relatively high at baseline in both the control (744 median MET minutes per week IQR 99 to 1740) and intervention (605 MET minutes per week IQR 177 to 2079) groups. Both groups increased their self-reported physical activity between baseline and three months. However there was no evidence of a significant difference in the change between the groups (mean difference intervention vs. control -145 MET minutes per week 95% CI -636 to 347). There was no significant change in the hours spent sitting between the groups at follow-up (mean difference -0.9 hours 95% CI -2.6 to 0.8).

### Adverse events

There were no serious adverse events related to the trial. Participants in the intervention group (n = 74) were asked whether self-weighing affected their mood, the mean mood score was 5.0 (2.4) which represents no difference to mood. Self-weighing did not affect the way that participants felt about their body, mean score 4.7 (2.4). A score of five is equal to no difference.

## Discussion

The instruction to weigh oneself daily and support to do so did not lead to greater weight loss than was achieved by participants who received no such instruction. Most people in the intervention group weighed themselves nearly every day and adherence to self-weighing was not associated with greater weight loss. There was little evidence that instructing daily self-weighing prompted greater uptake of behavioural strategies to control weight including more physical activity.

### Strengths and limitations

Obesity is associated with socio-economic disadvantage and 92% of participants lived in neighbourhoods that were poorer than the average for the UK. Participants were representative of those invited in age, gender and IMD. The proactive approach to recruit people who were obese resulted in recruiting men, people in socio-economic deprivation, and people in minority ethnic groups, which shows that such people who are often under-represented in weight loss trials do want support.

We had very few exclusion criteria which led to recruitment of a population that had a range of long-term health conditions, and we could infer this intervention may not be effective for these people. Participants may not have had the capability to change their behaviour which is proposed to be one of three key components that are needed for change to occur [29]. This may be due to their health conditions and perhaps participants required more support than we gave in this brief intervention. We did not measure any psychological constructs such as dietary restraint, disinhibition and weight locus of control (WLOC) which have been shown to be associated with self-weighing [12,30]. Dietary restraint may increase as self-weighing provides feedback and primes the person about cues to eat and thus may increase cognitive awareness and promote restraint [12,31]. Disinhibition may also decrease as if people are weighing daily they can keep on track with their weight goals [31]. If people have higher weight locus of control they may interpret the scale readings and feel more able to make changes to their diet and physical activity. Thus self-weighing may be more effective if participants have higher WLOC as they believe they have the control to manage their weight. Future studies should include these to better understand the mechanisms of effect for self-weighing.

A study strength was that we were able to investigate self-weighing as an isolated strategy by utilising an approach in which both groups received a minimal “sham” intervention that we know is ineffective [6]. This ensured blinding and that we had good follow-up rates in the control group and therefore reduced bias. We used self-reported weight (n = 15) when an objective measure could not be obtained, however a sensitivity analysis showed that removing self-reported data from analyses did not significantly change the results. The study was short and we had intended to follow-up participants at 12 months but when no effect was found at three months it was decided not to undertake further follow-up since differences in weight loss trials tend to decrease over time.

We instructed participants to weigh themselves daily to habitualise self-weighing. Participants weighed themselves on average 73.1 of the 90 days of the study. We did use scales that recorded whether participants weighed themselves but there were technical failures. Objective measures are likely to incr\ease the reliability of self-report measures, but we found little evidence that diaries had been retrospectively completed at follow-up to please the investigators. However, only 55% of participants returned their record card and therefore we used a conservative assumption that those who did not were not weighing themselves.

We measured whether self-weighing affected how participants felt at three months follow-up in the intervention group only. However we did not measure the change in mood/feelings from baseline in both groups which would have been more accurate and should be completed in future studies.

### Results in the context of other studies

These results are similar to two previous RCTs [14,15]. A small trial of 23 obese participants were randomised to weigh themselves daily or advised not to weigh themselves [15]. The control group had greater weight loss than the intervention group (5.9 vs. 4.6 kg) but the difference was not significant [15]. Participants in the control group were weighed before the group meetings therefore the effect of self-weighing may have been reduced. The second trial involved therapeutic interviews/consultations and the intervention group participants were additionally instructed to weigh themselves four times per day and record the weights on a chart [14]. At the end of the interviews/consultations there were no significant differences between the groups, however two years later the intervention group had maintained significantly greater weight loss than controls (14.9 vs. 7.8 kg). This result might suggest self-weighing could be more effective for weight maintenance than weight loss as found in other studies [16,32,33].

Participants may need additional support, beyond simply being instructed to weigh themselves in the early stages of weight loss as they may need to acquire other tools to manage their weight. Self-weighing relies upon participants having the motivation, capability and opportunity to enact changes to their diet and activity in response to feedback on their weight. Self-weighing is a tool aimed primarily at providing feedback to enact what people know they should already do. In our trial participants may have lacked this knowledge or the capability to incorporate these other behavioural strategies into their daily life and is perhaps why no effect was found.

Michie and colleagues examined the effectiveness of behaviour change techniques for alcohol reduction, physical activity and healthy eating and found that self-monitoring was associated with greater effectiveness [10,11]. However, when adding other self-control techniques (prompt intention formation, prompt specific goal setting, prompt review of behavioural goals and provide feedback of performance) the effect size increased. However it is not always possible (due to costs and time) to implement interventions with multiple techniques and thus we isolated the effect of self-weighing. In this trial simply asking participants to weigh themselves daily was insufficient, however multi-component interventions that include self-weighing provide evidence that self-weighing is an effective component for weight management [13,16]. We now need to add techniques to self-weighing to improve effectiveness and also ensure that the intervention could still be practically implemented to reach more people. No adverse effects of daily weighing were found and is line with previous research [34].

## Conclusions

Previous systematic reviews have shown that self-weighing is associated with better weight control and some trials have shown that elaborate interventions centred on self-weighing are also effective [13,16,21,31]. This trial is the first to isolate the effectiveness of the instruction to self-weigh together with a simple record card. There was no evidence it neither increased weight loss nor evidence that greater adherence to daily weighing was associated with greater weight loss. Advice to weigh oneself daily is ineffective as a sole strategy. It is important to add behavioural techniques or components incrementally to find a brief intervention that may be effective for promoting weight loss in primary care.

## References

[1] Sciamanna CN, Tate DF, Lang W, Wing RR (2000). Who reports receiving advice to lose weight?: Results from a multistate survey. Arch Intern Med.

[2] Jackson JE, Doescher M, Saver B, Hart LG (2005). Trends in professional advice to lose weight among obese adults, 1994 to 2000. J GEN INTERN MED.

[3] Leverence RR, Williams RL, Sussman A, Crabtree BF (2007). Am J Prev Med.

[4] Jackson SE, Wardle J, Johnson F, Finer N, Beeken RJ (2013). The impact of a health professional recommendation on weight loss attempts in overweight and obese British adults: a cross-sectional analysis. BMJ Open.

[5] Mehrotra C, Naimi T, Serdula M, Bolen J, Pearson K (2004). Arthritis, body mass index and professional advice to lose weight: implications for clinical medicine and public health. Am J Prev Med.

[6] Jolly K, Lewis A, Beach J, Denley J, Adab P, Deeks JJ, Daley A, Aveyard P (2011). Comparison of range of commercial or primary care led weight reduction programmes with minimal intervention control for weight loss in obesity: lighten up randomised controlled trial. BMJ.

[7] Jebb SA, Ahern AL, Olson AD, Aston LM, Holzapfel C, Stoll J, Amann-Gassner U, Simpson AE, Fuller NR, Pearson S, Lau NS, Mander AP, Hauner H, Caterson ID (2011). Primary care referral to a commercial provider for weight loss treatment versus standard care: a randomised controlled trial. Lancet.

[8] Nicklas JM, Huskey KW, Davis RB, Wee CC (2012). Successful weight loss among obese U.S. adults. Am J Prev Med.

[9] Wardle J, Johnson F (2002). Weight and dieting: examining levels of weight concern in British adults. Int J Obes.

[10] Michie S, Whittington C, Hamoudi Z, Zarnani F, Tober G, West R (2012). Identification of behaviour change techniques to reduce excessive alcohol consumption. Addiction.

[11] Michie S, Abraham C, Whittington C, McAteer J, Gupta S (2009). Effective techniques in healthy eating and physical activity interventions: a meta-regression. Health Psychol.

[12] Pacanowski CR: *Effects of self-weighing and visual feedback on weight control in adults.* Cornell University; 2013. https://dspace.library.cornell.edu/bitstream/1813/34400/1/crp56.pdf.

[13] Steinberg DM, Tate DF, Bennett GG, Ennett S, Samuel-Hodgea C, Ward DS (2013). The efficacy of a daily self-weighing weight loss intervention using smart scales and email. Obesity.

[14] Fujimoto K, Sakata T, Etou H, Fukagawa K, Ookum AK, Terada K, Kurata K (1992). Charting of daily weight pattern reinforces maintenance of weight reduction in moderately obese patients. American Journal of Medical Science.

[15] Heckerman CL, Brownell KD, Westlake RJ (1978). Self and external monitoring of weight. Psychol Rep.

[16] Wing RR, Tate DF, Gorin AA, Raynor HA, Fava JL (2006). A self-regulation program for maintenance of weight loss. N Engl J Med.

[17] Boutelle K (2006). Weighing the evidence: benefits of regular weight monitoring for weight control. Journal of Nutrition Education and Behaviour.

[18] Lally P, Chipperfield A, Wardle J (2008). Healthy habits: efficacy of simple advice on weight control based on a habit-formation model. Int J Obes.

[19] Department of Health (2006). Your weight your health - how to take control of your weight. Book Your Weight Your Health - how to Take Control of Your Weight.

[20] NICE (2006). Obesity Guidance on the prevention, identification, assessment and management of overweight and obesity in adults and children. Book Obesity Guidance on the Prevention, Identification, Assessment and Management of Overweight and Obesity in Adults and Children.

[21] VanWormer JJ, French SA, Pereira MA, Welsh EM (2008). The impact of regular self-weighing on weight management: a systematic literature review. International Journal of Behavioural Nutrition and Physical Activity.

[22] Lally P, Gardner B (2011). Promoting habit formation. Health Psychology Review.

[23] Michie S, Ashford S, Sniehotta FF, Dombrowski SU, Bishop A, French DPPH (2011). A refined taxonomy of behaviour change techniques to help people change their physical activity and healthy eating behaviours – the CALO-RE taxonomy. Psychol Health.

[24] Kanfer FH (1970). Self-monitoring: methodological limitations and clinical applications. Journal of Consulting and Clinical Applications.

[25] Noble M, McLennan D, Wilkinson K, Whitworth A, Barnes H, Dibben C (2008). The English Indices of Deprivation 2007. Book The English Indices of Deprivation 2007.

[26] Sciamanna CN, Kiernan M, Rolls BJ, Boan J, Stuckey H, Kephart D, Miller CK, Jensen G, Hartman TJ, Loken E, Hwang KO, Williams RJ, Clark MA, Schubart JR, Nezu AM, Lehman E, Dellasega C (2011). Practices associated with weight loss versus weight-loss maintenance results of a national survey. Am J Prev Med.

[27] Craig C, Marshall A, Sjöström M, Bauman AE, Booth ML, Ainsworth BE, Pratt M, Ekelund U, Yngve A, Sallis JF, Pekka OJ (2003). International physical activity questionnaire: 12-country reliability and validity. Med Sci Sports Exerc.

[28] Jette M, Sidney K, Blumchent G (1990). Metabolic equivalents (METS) in exercise testing, exercise prescription and evaluation of functional capacity. Clin Cardiol.

[29] Michie S, van Stralen MM, West R (2011). The behaviour change wheel: a new method for characterising and designing behaviour change interventions. Implement Sci.

[30] Strimas R, Dionne MM (2010). Differential effects of self-weighing in restrained and unrestrained eaters. Personal Individ Differ.

[31] Butryn ML, Phelan S, Hill JO, Wing RR (2007). Consistent self-monitoring of weight: a key component of successful weight loss maintenance. Obesity.

[32] Van Wormer JJ, Linde JA, Harnack LJ, Stovitz SD, Jeffery RW (2011). Self-weighing frequency is associated with weight gain prevention over two years among working adults. International Journal of Behavioural Medicine.

[33] Madigan CD, Aveyard P, Jolly K, Denley J, Lewis A, Daley A: **Regular self-weighing to promote weight maintenance after intentional weight loss: a quasi randomised controlled trial.** *Journal of Public Health* 2013, epub. 10.1093/pubmed/fdt061 23753256

[34] Steinberg DM, Tate DF, Bennett GG, Ennett S, Samuel-Hodge C, Ward DS (2014). Daily self-weighing and adverse psychological outcomes: a randomised controlled trial. Am J Prev Med.

